# Association of n-3 polyunsaturated fatty acid intakes with juvenile myopia: A cross-sectional study based on the NHANES database

**DOI:** 10.3389/fped.2023.1122773

**Published:** 2023-04-17

**Authors:** Zixiu Zhou, Sizhen Li, Qingsong Yang, Xiaodong Yang, Yating Liu, Kuanxiao Hao, Shanshan Xu, Na Zhao, Pengjie Zheng

**Affiliations:** Department of Ophthalmology, Nanjing Tongren Hospital, School of Medicine, Southeast University, Nanjing, China

**Keywords:** n-3 PUFA, myopia, juvenile, NHANES, cross-sectional study

## Abstract

**Aim:**

Inflammation is involved in the development of myopia. n-3 polyunsaturated fatty acids (n-3 PUFAs) have vasodilating and anti-inflammatory effects, which may be involved in controlling myopia. It is of great significance to explore the relationship between n-3 PUFA intakes and juvenile myopia in order to control and alleviate myopia among teenagers through dietary intervention.

**Methods:**

Sociodemographic data, information of nutrient intakes, cotinine, PUFAs, and eye refractive status of 1,128 juveniles were extracted from the National Health and Nutrition Examination Survey (NHANES) database in this cross-sectional study. PUFAs contained total polyunsaturated fatty acid (TPFAs), alpha-linolenic acid, octadecatetraenoic acid, eicosapentaenoic acid (EPA), docosapentaenoic acid (DPA), and docosahexaenoic acid (DHA). Covariates were screened by comparison among groups of normal vision, low myopia, and high myopia. The association between n-3 PUFA intakes and the risk of juvenile myopia was evaluated using univariate and multivariate logistic regression analyses with odds ratios (ORs) and 95% confidence intervals (CIs).

**Results:**

Among the juveniles, 788 (70.68%) had normal vision, 299 (25.80%) had low myopia, and 41 (3.52%) had high myopia. There were significant differences in average EPA and DHA intakes among the three groups, and mean DPA and DHA intakes in the normal vision group were lower than those in the low myopia group (*P *< 0.05). After adjustment for age, gender, TPFAs, and cotinine, a high dietary intake of EPA (≥11 mg/1,000 kcal) in juveniles seemed to be associated with the risk of high myopia (OR = 0.39, 95% CI: 0.18–0.85), while no significant associations were identified between n-3 PUFA intakes and the risk of low myopia.

**Conclusion:**

A high dietary intake of EPA may be associated with a decreased risk of high myopia among juveniles. A further prospective study is needed to validate this observation.

## Introduction

Myopia is a complex disease caused by genetic and environmental factors, which occurs in childhood and early adulthood, and has become a major cause of blindness (1). Myopia is a major public health issue worldwide and is expected to affect 49.8% of the global population in 2050, of which 20% could develop high myopia (2). Myopia-associated complications such as cataract, glaucoma, and retinal detachment can have serious impacts on juveniles and cause a great socioeconomic burden (3–5). Therefore, it is of great importance to explore effective interventions to prevent and alleviate juvenile myopia.

Dietary intervention may be an effective and safe way for the prevention and control of myopia in juveniles, in addition to pharmacological and optical tools. A recent study based on untargeted metabolomics showed that the amounts of serum fatty acid (FA) metabolites were reduced in myopic human subjects, and, in particular, the levels of serum docosahexaenoic acid (DHA) were significantly lower (6). These results indicated that FA metabolism may play a role in the occurrence and development of myopia. The effects of DHA and eicosapentaenoic acid (EPA) on promoting vascular smooth muscle cell relaxation and vasodilation have been identified to be associated with the inhibition of myopia development (7, 8). In the development of myopia, significant thinning of choroidal thickness and a decrease in choroidal blood perfusion are thought to cause local hypoxia in the retina and sclera, promoting axial elongation and subsequently accelerating myopia development (9–12). A study reported that EPA and its metabolites were associated with inhibition of choroidal thinning and myopia progression in mice (13). An *in vitro* experiment showed that DHA or EPA could antagonize hypoxia-induced myofibroblast trans-differentiation in cultured human scleral fibroblasts (2). In addition, oral n-3 polyunsaturated fatty acid (n-3 PUFA) supplementation partially alleviated choroidal blood perfusion induced by near work in human subjects (2). Herein, we speculated that higher dietary intakes of n-3 PUFAs may be protective against myopia.

The aim of this study was to explore the association between dietary intakes of n-3 PUFAs, including alpha-linolenic acid (ALA), octadecatetraenoic acid (SDA), EPA, docosapentaenoic acid (DPA), and DHA, and the risk of juvenile myopia to provide some dietary references for the clinical prevention and treatment of juvenile myopia.

## Methods

### Study design and population

Data for this cross-sectional study were extracted from the National Health and Nutrition Examination Survey (NHANES) database, a representative survey research program to assess the health and nutritional status of adults in the United States of America. Regular data collection of the NHANES is carried out on approximately 5,000 persons from 15 areas since 1999 and examined in 2-year periods. The NHANES is a multistage stratified sampling database, and our statistical analyses were based on a public link address (https://wwwn.cdc.gov/nchs/nhanes/). Permission was obtained to use the data in this study: https://www.cdc.gov/nchs/data_access/restrictions.htm.

A total of 2,011 juveniles aged 12–19 years who underwent refraction measurements were initially included. Information on their n-3 PUFA and energy intakes was obtained. Participants without information on refraction measurements, FAs, cotinine, energy intake, and history of eye surgery were excluded. Since the NHANES survey protocol was approved by the Ethics Review Board (ERB) of the National Center for Health Statistics (NCHS), data were de-identified, and informed consent was obtained from all participants; approval from our Institutional Review Board was not required for this study.

### n-3 PUFA measurement and the definition of myopia

In the NHANES, the method modified by Lagerstedt et al. was used to determine FAs (14). A total of 30 dietary FAs were quantitated. In brief, hexane along with an internal standard solution were used for the total extraction and recovery of FA. The extract was derivatized to form pentafluorobenzyl esters and injected into a capillary gas chromatograph column. The lower limit of detection of EPA, DHA, and DPA was 0.79, 1.84, and 0.24 µmol/L, respectively. FAs were detected using an Agilent 7890 B gas chromatograph equipped with a flame ionization detector and an Agilent DB-WAX capillary column (30 m × 0.25 μm × 0.25 μm) (15). In this study, the intakes of total polyunsaturated fatty acids (TPFAs) and n-3 PUFAs including ALA (g), SDA (g), EPA (g), DPA (g), and DHA (g) were calculated as energy density (kcal) separately (mg/1,000 kcal).

The refractive status of the eye was objectively assessed by non-cycloplegic objective auto-refractor (Nidek ARK-760) measurements in the vision database. Recorded refractive errors (median of three repeated measures) were included in the analyses only if a confidence of at least 5 (ranging from 1 to 9) was achieved at the time of measurement. Spherical equivalent refractions (SERs, i.e., average of the refractions in two principal meridians) were calculated from the right eye data of all included juveniles. Given that no cycloplegic agent was used in measuring refractive errors, this more conservative definition of myopia was employed to avoid misclassification of myopia. In this study, myopia was defined as an SER value ≤−1.0 diopter (D), and high myopia was defined as an SER value ≤−5.0 D. Participants were divided into three groups: normal vision (SER > −1.0), low myopia (SER ≤ −1.0), and high myopia (SER ≤ −5.0 D) (16).

### Covariates

Plasma PUFA levels may be influenced by gender, age, race, education level, family poverty impact ratio (PIR), activity, body mass index (BMI) (17, 18), prescription medication use, and dietary supplement use, which were considered potential confounders and adjusted for in this study (19). BMI was calculated as weight (kg) divided by the square of height (m^2^). Dietary nutrient and energy intakes were estimated from two 24-h dietary recall interviews by the United States Department of Agriculture (USDA) automated multiple-pass method (AMPM). The first interview was conducted in person, and a subsequent interview was conducted 3–10 days after the first one *via* a phone call. A dietary supplement questionnaire, as part of the NHANES household interview, was used to assess dietary supplements such as vitamins and minerals. The consumption frequency, duration, and dosage of each dietary supplement were recorded over the prior 30 days, which can be used to calculate the average daily intake of nutrients. The nutrient content of foods was determined by using the relevant Food and Nutrient Database for Dietary Studies (FNDDS) for each NHANES release.

### Statistical analysis

The data in this study were weighted, so continuous data were expressed as mean and standard error (SE) in the large weighted sample. Analysis of variance (ANOVA) was used to reflect the differences between groups. Categorical data were represented as [*N* (%)], and the chi-square test was used to analyze the differences between groups. Univariate and multivariate logistic regression analyses were used to explore the association between n-3 PUFA intakes and the risk of juvenile myopia. Model 1 was a crude model, Model 2 was adjusted for age and sex, and Model 3 was adjusted for age, gender, TPFA, and cotinine. The evaluation indicators were odds ratios (ORs) and 95% confidence intervals (CIs). All statistical tests were two-sided. *P *< 0.05 was considered significant. Analyses were performed with SAS 9.4 (SAS Institute Inc., Cary, NC, United States). Multiple imputation and sensitivity analysis were performed on missing data, as shown in Supplementary Table S1.

## Results

### Characteristics of juveniles

**Figure 1:**
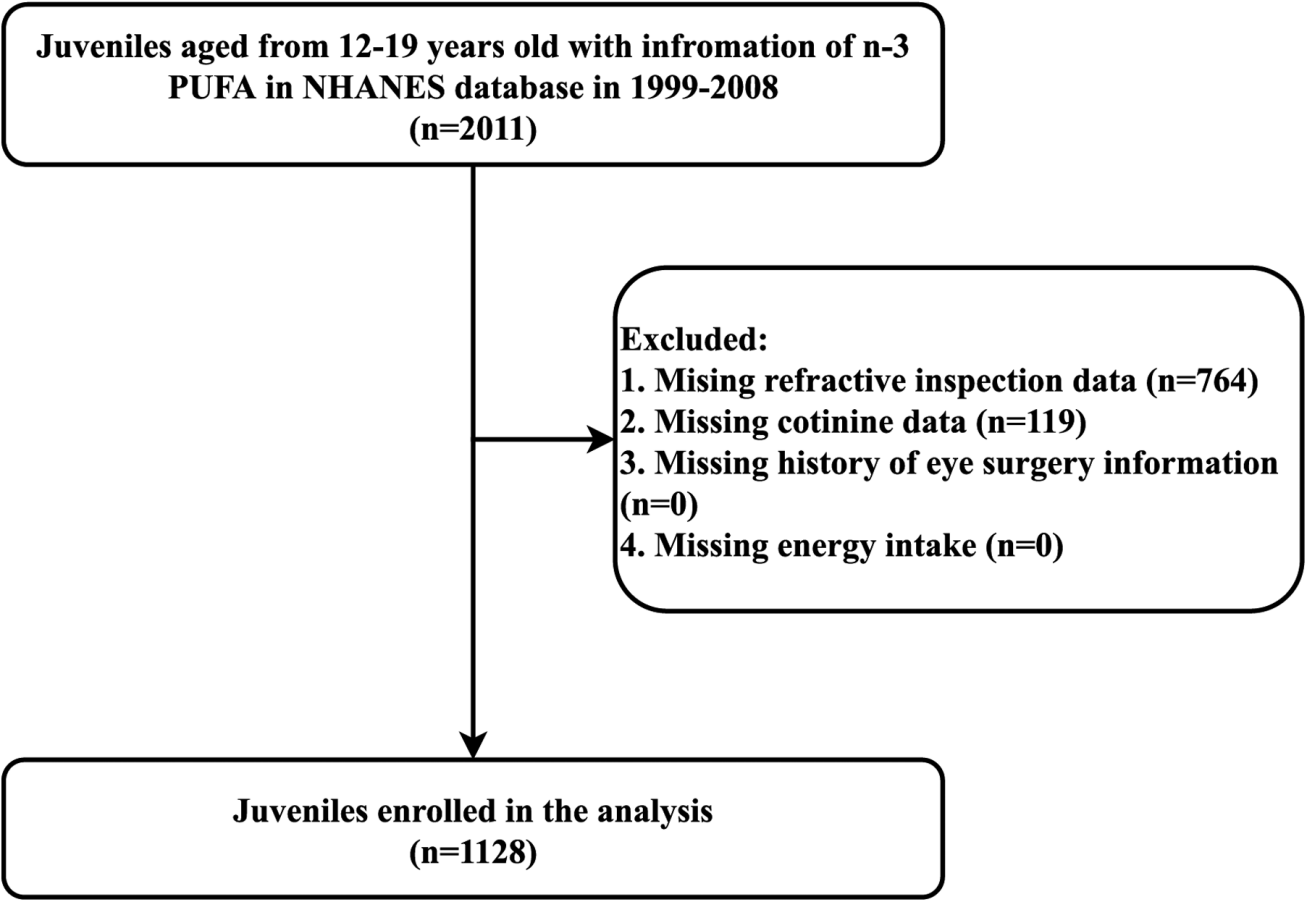
The flow chart of data screening.

Figure 1 shows the flow chart of data screening. A total of 2,011 juveniles aged 12–19 years with information on n-3 PUFA intakes from the NHANES database between 1999 and 2008 were initially included. After excluding those with missing information on refraction measurements (*n* = 764) and cotinine (*n* = 119), 1,128 juveniles were finally eligible for this study.

Table 1 shows the characteristics of juveniles with normal vision, low myopia, and high myopia. Among 1,128 juveniles, 788 (70.68%) had normal vision, 299 (25.80%) had low myopia, and 41 (3.52%) had high myopia. The average age of the participants was 15.47 ± 0.08 years, and the number of males and females was equal (both *n* = 564). There were significant differences in the average cotinine level and the axis of refractive astigmatism in right eyes among the normal vision, low myopia, and high myopia groups (all *P *< 0.05). Significant differences were observed in the intakes of vitamin C, protein, and total fat between the normal vision group and the high myopia group, and between the low myopia group and the high myopia group (all *P *< 0.05).

**Table 1:** Characteristics of juveniles with normal vision, low myopia, and high myopia.

Variables	Total (*n* = 1,128)	High myopia (*n* = 41)	Low myopia (*n* = 299)	Normal vision (*n* = 788)	Statistics	*P*
Age, years, mean ± SE	15.47 ± 0.08	15.85 ± 0.49	15.59 ± 0.20	15.41 ± 0.11	F = 0.47	0.624
Gender, *n* (%)					*χ*^2 ^= 2.158	0.340
Female	564 (49.01)	20 (36.78)	153 (52.34)	391 (48.41)		
Male	564 (50.99)	21 (63.22)	146 (47.66)	397 (51.59)		
Ethnicity, *n* (%)					*χ*^2 ^= 4.187	0.840
Mexican American	334 (12.57)	11 (9.94)	98 (14.79)	225 (11.89)		
Non-Hispanic Black	426 (18.42)	17 (18.94)	110 (18.42)	299 (18.40)		
Non-Hispanic White	260 (56.47)	9 (62.91)	60 (52.32)	191 (57.67)		
Other Hispanic	63 (6.45)	3 (4.84)	18 (7.88)	42 (6.01)		
Other Race	45 (6.08)	1 (3.37)	13 (6.59)	31 (6.03)		
Education level, *n* (%)					*χ*^2 ^= 3.144	0.208
Less than 9th grade	486 (41.50)	13 (27.92)	129 (37.96)	344 (43.47)		
More than high school	642 (58.50)	28 (72.08)	170 (62.04)	444 (56.53)		
PIR, mean ± SE	2.65 ± 0.08	2.89 ± 0.44	2.76 ± 0.15	2.59 ± 0.10	F = 0.51	0.601
HH Ref person education level, *n* (%)					*χ*^2 ^= 11.129	0.084
9th–11th grade	235 (13.60)	6 (6.00)	61 (12.28)	168 (14.46)		
High school grade	273 (25.76)	7 (11.38)	71 (23.27)	195 (27.38)		
Less than 9th grade	137 (6.53)	3 (2.89)	36 (5.77)	98 (6.99)		
Some college or above	483 (54.11)	25 (79.72)	131 (58.67)	327 (51.17)		
BMI, kg/m^2^, mean ± SE	23.45 ± 0.23	23.79 ± 1.16	23.68 ± 0.32	23.36 ± 0.30	F = 0.26	0.770
Vigorous activity over past 30 days, *n* (%)					*χ*^2 ^= 0.920	0.631
No	350 (28.45)	11 (32.63)	86 (25.70)	253 (29.24)		
Yes	778 (71.55)	30 (67.37)	213 (74.30)	535 (70.76)		
Moderate activity over past 30 days, *n* (%)					*χ*^2 ^= 1.192	0.551
No	415 (35.70)	15 (46.02)	108 (33.92)	292 (35.84)		
Yes	713 (64.30)	26 (53.98)	191 (66.08)	496 (64.16)		
Cotinine, ng/mL, Mean ± SE	16.68 ± 2.07	18.23 ± 10.27	7.98 ± 2.68	19.78 ± 2.62	F = 4.63	0.013
Vitamin A, μg, mean ± SE	771.53 ± 92.20	867.37 ± 98.11	689.95 ± 78.23	796.54 ± 125.92	F = 0.94	0.394
Vitamin C, mg, mean ± SE	102.30 ± 6.31	239.68 ± 111.63	105.45 ± 8.37^a^	94.32 ± 5.06^a^	F = 1.52	0.226
Vitamin E as alpha-tocopherol, mg, mean ± SE	8.39 ± 0.37	8.58 ± 0.82	8.23 ± 1.01	8.43 ± 0.40	F = 0.04	0.963
Energy intake, kcal, mean ± SE	2,612.22 ± 56.95	2,999.57 ± 243.02	2,565.60 ± 130.83	2,609.95 ± 77.03	F = 1.32	0.272
Protein intake, g, mean ± SE	97.87 ± 2.22	118.83 ± 13.93	97.82 ± 4.98^a^	96.84 ± 3.06^a^	F = 1.19	0.311
Carbohydrate, g, mean ± SE	329.23 ± 6.96	348.21 ± 40.89	320.05 ± 14.91	331.63 ± 9.53	F = 0.29	0.751
Total fat intake, g, mean ± SE	101.07 ± 2.80	126.04 ± 10.33	100.82 ± 7.15^a^	99.91 ± 3.35^a^	F = 2.85	0.064
Zinc, mg, mean ± SE	13.36 ± 0.43	14.79 ± 1.41	12.96 ± 0.70	13.43 ± 0.61	F = 0.72	0.491
Magnesium, mg, mean ± SE	291.30 ± 6.06	316.53 ± 28.35	296.68 ± 16.44	288.08 ± 7.51	F = 0.51	0.604
Selenium, μg, mean ± SE	138.69 ± 2.86	144.18 ± 14.42	137.90 ± 7.99	138.71 ± 3.88	F = 0.08	0.922
Copper, mg, mean ± SE	1.39 ± 0.04	1.43 ± 0.13	1.41 ± 0.10	1.38 ± 0.04	F = 0.07	0.937
Right axis of refractive astigmatism, mean ± SE	79.45 (3.38)	91.28 (10.94)	91.09 (5.05)	74.62 (4.04)^b^	F = 4.20	0.019
Left axis of refractive astigmatism, mean ± SE	94.58 (2.95)	87.62 (13.44)	93.29 (4.21)	95.40 (4.34)	F = 0.14	0.870
Right keratometry power flat curve, D, mean ± SE	43.39 (0.25)	43.16 (0.46)	43.51 (0.11)	43.35 (0.33)	F = 0.39	0.675
Left keratometry power flat curve, D, mean ± SE	43.47 (0.19)	43.62 (0.40)	43.54 (0.11)	43.44 (0.25)	F = 0.11	0.895
Right keratometry power steep curve, D, mean ± SE	44.42 (0.25)	45.24 (0.72)	44.58 (0.12)	44.33 (0.33)	F = 0.88	0.420
Left keratometry power steep curve, D, mean ± SE	44.40 (0.20)	44.64 (0.43)	44.60 (0.11)	44.31 (0.26)	F = 0.81	0.450

SE, standard error; PIR, poverty income ratio; BMI, body mass index; HH Ref person education level, tutorial education level; *χ*^2^, chi-square test; F, analysis of variance.

^a^ Compared with high myopia group (*P *< 0.05).

^b^ Compared with low myopia group (*P *< 0.05).

### n-3 PUFA intakes among juveniles with normal vision, low myopia, and high myopia

Table 2 presents a comparison of n-3 PUFA intakes among the three groups. In the unprocessed data, the differences in EPA (*P *= 0.049) and DHA (*P *= 0.047) intakes were statistically significant among the normal vision, low myopia, and high myopia groups. Compared with juveniles with low myopia, those with normal vision consumed significantly less DPA (0.03 vs. 0.04 g) and DHA (0.16 ± 0.01 vs. 0.20 ± 0.03 g) intakes. Moreover, in terms of energy density, the mean intakes of EPA (*P *= 0.006) and DHA (*P *= 0.002) were also significantly different among the three groups. The average intakes of EPA (49.50 ± 7.06 vs. 21.65 ± 8.45 g) and DHA (88.73 ± 12.18 vs. 44.25 ± 10.47 g) in juveniles with low myopia were significantly higher than those in juveniles with high myopia. The intakes of DPA and DHA in juveniles with normal vision were lower than those in juveniles with low myopia (*P *< 0.05).

**Table 2:** Comparison for n-3 PUFA intake among juveniles with normal vision, low myopia, and high myopia.

Variables	Total (*n* = 1,128)	High myopia (*n* = 41)	Low myopia (*n* = 299)	Normal vision (*n* = 788)	Statistics	*P*
Unprocessed data						
TPFA, g, mean ± SE	22.73 ± 0.66	24.29 ± 2.67	23.05 ± 1.77	22.53 ± 0.75	F = 0.21	0.812
n-3 PUFA, g, mean ± SE	2.26 ± 0.06	2.36 ± 0.27	2.33 ± 0.17	2.23 ± 0.07	F = 0.18	0.833
ALA, g, mean ± SE	1.92 ± 0.06	2.11 ± 0.27	1.92 ± 0.16	1.90 ± 0.07	F = 0.28	0.760
SDA, g, mean ± SE	0.04 ± 0.00	0.03 ± 0.01	0.04 ± 0.01	0.04 ± 0.00	F = 0.50	0.610
EPA, g, mean ± SE	0.10 ± 0.01	0.06 ± 0.02	0.11 ± 0.02	0.09 ± 0.01	F = 3.15	0.049
DPA, g, mean ± SE	0.04 ± 0.00	0.03 ± 0.01	0.04 ± 0.00	0.03 ± 0.00^a^	F = 1.05	0.355
DHA, g, mean ± SE	0.17 ± 0.01	0.12 ± 0.03	0.20 ± 0.03	0.16 ± 0.01^a^	F = 3.18	0.047
Energy density						
TPFA, mg/1,000 kcal, mean ± SE	8,750.81 ± 161.94	7,719.91 ± 469.68	8,878.69 ± 268.58	8,755.47 ± 203.35	F = 2.33	0.105
N-3 PUFA, mg/1,000 kcal, mean ± SE	882.93 ± 19.23	769.11 ± 47.54	908.64 ± 34.34	879.22 ± 24.02	F = 3.01	0.055
ALA, mg/1,000 kcal, mean ± SE	730.94 ± 16.36	677.74 ± 56.00	735.00 ± 30.11	732.11 ± 21.06	F = 0.45	0.641
SDA, mg/1,000 kcal, mean ± SE	16.77 ± 0.97	13.29 ± 2.73	16.45 ± 2.03	17.06 ± 1.29	F = 0.94	0.394
EPA, mg/1,000 kcal, mean ± SE	43.30 ± 3.17	21.65 ± 8.45	49.50 ± 7.06^b^	42.11 ± 3.58	F = 5.59	0.006
DPA, mg/1,000 kcal, mean ± SE	16.13 ± 0.98	12.17 ± 2.55	18.96 ± 2.26	15.29 ± 1.13^a^	F = 2.65	0.077
DHA, mg/1,000 kcal, mean ± SE	75.80 ± 4.82	44.25 ± 10.47	88.73 ± 12.18^b^	72.65 ± 5.06^a^	F = 6.90	0.002

TPFA, total polyunsaturated fatty acid; SE, standard error; ALA, alpha-linolenic acid; SDA, octadecatetraenoic acid; EPA, eicosapentaenoic acid; DPA, docosapentaenoic acid; DHA, docosahexaenoic acid; F, analysis of variance.

n-3 PUFAs include ALA, SDA, EPA, DPA, and DHA.

^a^ Compared with low myopia group (*P *< 0.05).

^b^ Compared with high myopia group (*P *< 0.05).

### Association between n-3 PUFA intakes and the risk of juvenile myopia

Table 3 shows the association between the intakes of n-3 PUFAs (calculated by energy density) and the risk of juvenile myopia. After adjustment for covariates including age, gender, TPFA, and cotinine, a high dietary intake of EPA (≥11 mg/1,000 kcal) seemed to be associated with a decreased risk of high myopia (OR = 0.39, 95% CI: 0.18–0.85) in juveniles. However, no significant association was found between n-3 PUFA intakes and the risk of low myopia. Additionally, the intake of EPA ≥11 mg/1,000 kcal was not associated with the risk of low myopia (OR = 1.21, 95% CI: 0.79–1.87, *P *= 0.374).

**Table 3:** Association between n-3 PUFA intakes and the risk of juvenile myopia.

Variables	Model 1		Model 2		Model 3	
	OR (95% CI)	*P*	OR (95% CI)	*P*	OR (95% CI)	*P*
High myopia vs. normal vision						
Energy density						
n-3 PUFA						
<759.65	Ref		Ref		Ref	
≥759.65	0.51 (0.23–1.12)	0.092	0.51 (0.24–1.07)	0.076	0.44 (0.19–1.02)	0.056
ALA						
<637.85	Ref		Ref		Ref	
≥637.85	0.96 (0.33–2.84)	0.946	0.97 (0.32–2.91)	0.952	0.91 (0.24–3.39)	0.886
SDA						
<7.51	Ref		Ref		Ref	
≥7.51	0.82 (0.33–2.08)	0.678	0.82 (0.33–2.07)	0.678	0.83 (0.33–2.08)	0.684
EPA						
<11	Ref		Ref		Ref	
≥11	0.37 (0.17–0.81)	0.014	0.38 (0.17–0.84)	0.017	0.39 (0.18–0.85)	0.018
DPA						
<8.18	Ref		Ref		Ref	
≥8.18	1.12 (0.44–2.84)	0.811	1.10 (0.44–2.75)	0.844	1.10 (0.43–2.83)	0.846
DHA						
<31.4	Ref		Ref		Ref	
≥31.4	0.72 (0.25–2.08)	0.533	0.73 (0.25–2.15)	0.562	0.74 (0.25–2.18)	0.577
Low myopia vs. normal vision						
Energy density						
TPFA						
<803.55	Ref		Ref		Ref	
≥803.55	1.25 (0.86–1.83)	0.235	1.22 (0.83–1.79)	0.298	1.12 (0.64–1.96)	0.679
n-3 PUFA						
<759.65	Ref		Ref		Ref	
≥759.65	1.07 (0.74–1.56)	0.705	1.05 (0.72–1.53)	0.797	0.93 (0.55–1.56)	0.773
ALA						
<63.78	Ref		Ref		Ref	
≥63.78	0.90 (0.60–1.33)	0.584	0.91 (0.62–1.35)	0.648	0.93 (0.63–1.37)	0.720
SDA						
<0.75	Ref		Ref		Ref	
≥0.75	1.12 (0.76–1.66)	0.551	1.12 (0.76–1.66)	0.550	1.11 (0.74–1.66)	0.612
EPA						
<1.1	Ref		Ref		Ref	
≥1.1	1.27 (0.84–1.91)	0.255	1.26 (0.84–1.90)	0.264	1.21 (0.79–1.87)	0.374
DPA						
<0.82	Ref		Ref		Ref	
≥0.82	1.16 (0.78–1.72)	0.449	1.15 (0.78–1.71)	0.469	1.13 (0.75–1.71)	0.551
DHA						
<3.14	Ref		Ref		Ref	
≥3.14	1.15 (0.78–1.72)	0.475	1.15 (0.78–1.71)	0.477	1.15 (0.77–1.71)	0.504

TPFA, total polyunsaturated fatty acid; OR, odds ratio; CI, confidence interval; Ref, reference; ALA, alpha-linolenic acid; SDA, octadecatetraenoic acid; EPA, eicosapentaenoic acid; DPA, docosapentaenoic acid; DHA, docosahexaenoic acid.

n-3 PUFAs include ALA, SDA, EPA, DPA, and DHA.

Model 1: univariate logistic regression; Model 2: adjusted the age and gender; Model 3: adjusted the age, sex, TPFA, and cotinine.

## Discussion

This study explored the association between n-3 PUFA intakes and the risk of juvenile myopia, and showed that a high dietary intake of EPA, calculated by energy density, was associated with a reduced risk of high myopia in juveniles. However, no association was found between n-3 PUFA intakes and the risk of low myopia.

Previous studies on myopia improvement with n-3 PUFAs were mostly conducted based on experimental animal models, and few have been explored in humans. Pan et al. (2) demonstrated that n-3 PUFAs, especially DHA, inhibited myopia development and that a decrease in choroidal blood perfusion induced by near work was partially alleviated by dietary n-3 PUFAs in human young adults. Ong et al. (20) found that n-3 oral nutritional supplements could improve visual acuity recovery after photorefractive keratectomy (PRK), suggesting that it may be a beneficial adjunct therapy for PRK patients. Similarly, the research study by Goyal et al. (21) demonstrated supplementation with n-3 FAs had a positive influence on tear secretion in patients undergoing laser *in situ* keratomileusis. In experimental animal myopia models (10, 12) and humans (22, 23), choroidal thickness and choroidal blood perfusion were significantly decreased, and these changes might subsequently trigger scleral hypoxia and subsequent myopia (11). n-3 PUFAs are essential for the synthesis of eicosanoids such as prostaglandins (PGs), prostacyclin (PGI), hydroperoxytetraenoic acid, hydroxyeicosatetraenoic acid, and lipoxins (24), which are involved in several physiological actions, including pro/anti-inflammatory response, pro/antiplatelet aggregatory response, vasodilation, vasoconstriction, immune response, and cell growth and proliferation, and metabolism imbalance could lead to several conditions. In addition, the vasodilating role of n-3 PUFAs in the cardiovascular system (25) and its neuroprotective capacity could also be observed in the retina and optic nerve (26, 27). The current study assumed that n-3 PUFAs may be involved in the relief of myopia through anti-inflammatory and choroid vasodilatation mechanisms.

The energy density of a food can be defined as the amount of metabolizable energy per unit weight of a food (kcal/g), derived from the macronutrient and moisture content of the food (28). As the most and least energy-dense nutrients, fat (9 kcal/g) and water (0 kcal/g) are the primary determinants of energy density (29). In the United Kingdom, children with myopia progression consumed more protein and lower fat and carbohydrate than children without progression (30). In Hong Kong, myopic children were more likely to report lower protein and fat intakes (31). We found that both protein and total fat intakes were lower in juveniles with normal vision and low myopia than those in juveniles with high myopia. However, Li et al. (32) found no significant association between specific nutrients or food groups and incident myopia, indicating that diet may not be associated with myopia in children (9 years old). The inconsistent results may be due to differences in the race, geography, and dietary habits of the study population. Prospective cohort studies are needed to further explore the effect of the nutrients on myopia in children.

Our findings showed that a high EPA intake (calculated by energy density) was associated with a reduced risk of high myopia in juveniles, but not with low myopia. A possible explanation is that the influence of diet and other environmental factors may be limited, as genetic factors may have a greater contribution to the early development of refractive errors (33). The diet of an infant at the age of 6 months consists mostly of formula or breast milk, and solid foods are gradually introduced from 6 to 12 months of age. The lack of variety in infant diets may result in limited variation in nutrient intakes, which may explain the observed negative findings (34). Moreover, a study suggested that metabolic changes in n-3 PUFAs occurred during the initial stages of myopia development, so early intervention targeting the PUFAs could ameliorate the onset and progression of myopia (2). Early intervention is particularly important because children who have developed myopia are continually exposed to myopic stimuli, such as increased near work, under which myopia is more likely to progress toward sight-threatening, high myopia (35). The retina has a complex and effective recycling system that ensures conservation of retinal DHA levels even in the presence of prolonged dietary n-3 PUFAs deficiency (36). In an animal model of form deprivation myopia (FDM), the levels of DHA and EPA were increased in the serum and the sclera, but decreased in the retina. DHA is abundant in the retina and plays an important role in the synthesis of disk membranes (37). It may be that a decrease in the serum DHA level enhances adaptation of the retina to metabolic stress. We hypothesized that the lack of significant improvement of n-3 PUFA supplementation in patients with low myopia may be due to the body's own metabolic compensatory mechanism, while sensitivity may be reduced in high myopia populations. In any case, the metabolism and function of n-3 PUFAs in high myopia are very complex. Further investigations are needed to probe into the role of n-3 PUFAs in myopia progression and identify the mechanism of action to lay a solid foundation for the treatment of human myopia.

The association between dietary n-3 PUFA intakes and the risk of myopia was explored in juveniles, which partially filled the gap in the relevant field and provided some references for the control of myopia by n-3 PUFA supplementation. However, there were some limitations in the study. This is a cross-sectional study, and the retrospective nature of the data makes it unable to reveal a causal association. The sample size for high myopia was small and modifiable confounders may have contributed to the negative results. The mechanism underlying the intake of these diets was unclear in the NHANES database, and further studies should take their effects on myopia into consideration. In addition, dietary intakes examined by the NHANES only reflected current dietary patterns, so prospective cohort studies are needed to investigate the association between long-term high n-3 PUFA intake and the risk of myopia in juveniles.

## Conclusion

A high dietary intake of EPA may be associated with a decreased risk of high myopia among juveniles. Further prospective studies are needed to explore the impact of n-3 PUFAs on myopia risk reduction in order to relieve the social and economic burden and complications of myopia.

## Data Availability

Publicly available datasets were analyzed in this study. These data can be found here: NHANES database, https://wwwn.cdc.gov/nchs/nhanes/.
