# Supplementary material for: Association of n-3 polyunsaturated fatty acid intakes with juvenile myopia: A cross-sectional study based on the NHANES database
Source: Front Pediatr. 2023 Apr 17;11:1122773. doi: 10.3389/fped.2023.1122773 (PMC10150007; doi:10.3389/fped.2023.1122773)
Supplement: Supplementary file 1 [file Table1.docx]

**Table S1 Sensitivity analysis of missing variables.**

| Variables | Before imputation (n=1128) | After imputation (n=1128) | Statistics | *P* |
| --- | --- | --- | --- | --- |
| Family PIR, Mean±SE | 2.66 (0.08) | 2.65 (0.08) | t=1.65 | 0.104 |
| HH Ref Person Education Level, n (%) |  |  | χ^2^=0.057 | 0.997 |
| 9-11th Grade | 228 (13.58) | 235 (13.60) |  |  |
| High School Grad | 262 (25.73) | 273 (25.76) |  |  |
| Less Than 9th Grade | 129 (5.89) | 137 (6.53) |  |  |
| Some College or above | 469 (54.81) | 483 (54.11) |  |  |
| Body Mass Index, kg/m^2^, Mean±SE | 23.45 (0.23) | 23.45 (0.23) | t=-0.81 | 0.420 |
| Vigorous activity over past 30 days, n (%) |  |  | χ^2^=0.145 | 0.703 |
| No | 346 (28.34) | 350 (28.45) |  |  |
| Yes | 763 (71.66) | 778 (71.55) |  |  |
| Moderate activity over past 30 days, n (%) |  |  | χ^2^=0.056 | 0.812 |
| No | 406 (35.75) | 415 (35.70) |  |  |
| Yes | 701 (64.25) | 713 (64.30) |  |  |

PIR: poverty–income ratio, SE: standard error

T: t-test, χ^2^: chi-square test
